# What Shall Be Done with the Homicidal Crank*Abstract of paper read before Allegheny Medical Society, May 28, 1895.

**Published:** 1895-09

**Authors:** Theodore Diller

**Affiliations:** Pittsburg, Pa.


### WHAT SHALL BE DONE WITH THE HOMICIDAL CRANK.*

By THEODORE DILLER, M.D.,

Pittsburg, Pa.

A year or two ago a man about fifty years of age came into my office and said that he was constantly kept under hypnotic influence by a certain man who lived in the same village in which he resided. He stated that a couple of years previously he first became aware of a certain malign influence exerted over him by hearing voices which accused him of incompetence and certain crimes and misdemeanors. These voices, heard rather infrequently at first, visited him oftener as time passed on. He grew to believe that his secret thoughts were read; that all lack of success and mishaps and accidents of various kinds were due to some evil influence which was exerted over him. He told his wife and friends of this malign power to which he then believed he was constantly subjected, and when they would not agree with him in his ideas, conceived the belief that they, too, were dominated by the same evil influence which had rendered him powerless. He came to believe shortly before coming to see me that the evil influence dominating his whole life was exerted through hypnotism, and then naturally cast about to find the author of this strange and marvelous influence. He thought of several persons, and at first could not be sure which was the one on whom he should blame his troubles. But at the time he had called on me he was quite sure he had found the real author of all his misery. He named this person to me, and with flashing eye and angry expression denounced him soundly.

My explanation to him that all these ideas were vagaries of the mind brought about by an illness from which he was suffering, was, as you may readily suppose, not received.

I engaged him in conversation on indifferent subjects, and found that he was rational and intelligent when talking of these matters. When he had left the office, I at once wrote to his wife to call

*Abstract of paper read before Allegheny Medical Society, May 28, 1895.

upon me, which she did some few days later. I urged her as strongly as possible to place her husband in an asylum at once, as he was a very dangerous man and might at any time attempt to kill the man whom he believed was keeping him constantly under hypnotic influence. She refused to do this because her husband was "so sensible" in many ways. She doubted whether his "peculiar ideas" were enough to constitute insanity. The man came back several times, each time telling the same story. He, however, became convinced that I was unable to remove the hypnotic spell which was upon him, but sought my aid in helping him to have his Svengali arrested and placed in prison. He said he wished to act within the law, but that if he failed in this, he might be constrained to take the law in his own hands.

I have purposely related this case at some length, as it is an excellent type of many so-called "homicidal cranks," and illustrates well some difficulties encountered in dealing with them.

This man's wife regarded him as "peculiar," but not insane. Yet the possession of those very reasoning powers which made his wife hold that he was not insane, rendered him, with the delusions and hallucinations he possessed, all the more dangerous. The old idea among the laity that to constitute insanity there must be great emotional disturbance or entire dethronement of reasoning faculties still largely prevails. So it happens that friends of the chronic delusional lunatic or paranoiac who constitute most frequently the "homicidal crank" are unwilling, as in the instance cited, to have him detained in an asylum. But they are often willing to have him so committed after he has killed some one and an indignant community are demanding that the death sentence be passed upon him.

Last summer a mechanic, somewhat past middle age, was admitted to St. Francis Hospital, not so much because he was insane as because it was feared that he would commit murder. In my first interview with him, I was, as is often the case, unable to discover any evidence of insanity. Before seeing him again I learned of delusions he entertained, and was, at my second visit, easily able to learn the character of his insanity. For some months past he had believed that his wife was unfaithful. He stayed home from work frequently to watch her, but even then he was convinced that she carried on illicit relations with different men. Every man who came to the house for any purpose was suspected. He stated that as many as six men had intercourse with his wife in a single night, and moreover affirmed that his own daughters shielded her in her infidelity, and kept a large black monkey about the house which in some way assisted them. It was noticed shortly before the unfortunate man was brought to the hospital that he had put in repair and loaded an old pistol which was about the house, and in speaking to me afterwards he said that it had been his intention to shoot some man when he was quite sure of his guilt. This action very properly determined the family to place the unhappy man in the hospital.

Here we have a case of paranoia, another type of the "homicidal crank." I think it will be readily conceded he might easily commit homicide.

Another type of paranoia is that characterized by great exaltation of the ego, together with delusions of persecution. Here the subject wishes to lead the nation, reform a great city, create a new religion, offer an easy plan for the settlement of political difficulties, etc. Finding, of course, difficulties in attaining his end, he logically sets about to ascertain the obstruction, and this he often finds to be some individual of great prominence. Frequently his attention is called to the individual who stands in the way of his own greatness, and the success of a great cause by some public discussion or dispute in the newspapers which bring the said individual into great prominence. Thus Guiteau thought to unify a great party by killing President Garfield; Berkman sought to simplify the labor trouble by attempting to kill Chairman Frick; while Prendergast believed he would cleanse and promote the progress of Chicago by killing Mayor Harrison. At some time or other attempts on the lives of most European sovereigns have been attempted by the "homicidal crank." Indeed, it may be said of him as of death, that he loves a "shining mark."

What is to be done with him? The question may be divided into two parts.

1. Before he has attempted or committed homicide?

2. After he has attempted or committed homicide?

The first of these two questions presents the greater difficulties. In the first place, not all the so-called "cranks" are homicidal. Indeed, many of them, I believe, may be safely left at large undisturbed. But in general, I should say that those who entertain systematized delusions of suspicion or persecution, especially in connection with exaggerated ideas of self-importance, ought to be under restraint. How and by whom is this class to be sought out and by what legal process restrained? These are the vital questions in connection with the matter. Obviously, it would be impracticable for the authorities to appoint special examiners to go about seeking out the "cranks" in the community. But it seems to me that much could be done by members of our profession to educate the public to some intelligent understanding of the dangers to which society, particularly some prominent individuals in the same, are ever subject to while certain of these "cranks" remain at large. Many of them are brought to the physician's notice in some way or other; and in that large conception of our professional duties which clearly points that ministering to those actually sick, mentally or physically, does not by any means comprise our whole duty, but that it belongs to us to go farther and warn families and the public generally against mental and physical harm which may be threatening. With a clear understanding of the nature of these cases of chronic delusional, alcoholic, epileptic, and other insanities which may bring forth the homicidal crank, and with this broader view of our relations and duties to society, the physicians may do much to lessen the number of murders by "homicidal cranks" at large. Nor should we be discouraged if our efforts sometimes prove futile. In the first case I have reported, looking upon my duty to the public as of paramount importance, and in my unsuccessful efforts to get the man in an asylum beyond the reach of harming any one, I revealed so much of professional confidence as seemed to me might be necessary to accomplish that purpose. It is very probable, however, that in reality, I revealed nothing that was not already known. But I could easily conceive of cases which might arise where in the discharge of his duty to the public the physician might be called upon to reveal much of a private nature. In delicate or doubtful cases, however, he should seek counsel before doing so.

I believe that persons who, by their actions, seem to be mentally deranged and seek interviews at the White House, with the various governors and prominent individuals, and who haunt our courts and legislative and other bodies, should promptly be arrested and examined by competent physicians as to their mental condition.

If after such examination they are shown to be "cranks" of dangerous tendencies, they should by some appropriate legal process be remanded to the asylum. I believe this matter should be taken up and pushed vigorously. At very short intervals, the newspapers furnish accounts of the doings of "cranks" at the White House. Unless he commits some overt act, I believe he is not arrested nor are efforts made to institute any legal inquiries as to his sanity. While many of these "cranks" are doubtless harmless, one must believe that some would under certain circumstances commit homicide. Extraordinary measures have been taken to protect the president during the past few years against the "crank." It seems to me that in Washington the method of inquiry I have suggested could be most profitably instituted, for the capital of the nation draws, and will continue to draw, large numbers of "cranks" to it, largely because their delusions of greatness take a political complexion, and the seat of the government naturally becomes a sort of Mecca for them.

After he has committed a homicide, what shall be done with the "crank"? Certainly he should not be hung. I cannot indorse the Spartan method of Dr. Wm. A. Hammond, who, in the Guiteau trial, held that the prisoner was insane, yet should be hung. This view, however, is, I believe, held by a large part of every community. The homicide is usually dastardly, and the "crank" makes a certain show of reason and always spurns the idea that he is insane. These facts lead very generally to a popular and to a sometimes almost unanimous demand on the part of the public for his execution, some holding that he is sane or shamming insanity; others contending that his mental operations, while abnormal, did not prevent him from knowing the enormity of his crime and the difference between right and wrong. In short, the crime has been so revolting that the public does not wish to hear of arguments which will cheat them out of a victim to atone for the sacrifice of the slain. Both the men whose cases I have related knew right from wrong; both of them might have committed murder; and if they had, probably both would have admitted it was wrong and offered as excuse that it was necessary to protect themselves and families. What did the public gain by hanging the unfortunate paranoiacs Guiteau and Prendergast. Yet both these men, too, probably knew right from wrong. Happily this antiquated and unreasonable "right and wrong" test is, in the light of a higher and better medical jurisprudence, showing signs of decay. But it still shows great vigor. Hasten the day when it shall have disappeared altogether as a sufficient standard of sanity or insanity!

If the homicidal crank is not to be hung, what is to be done with him? Certainly the public has the best right to demand protection from him.

This protection would be afforded by sending him to an asylum, there to remain until cured, or he might be sent for a certain term of years (10–15–20) and as much longer as might be required for his cure. Sent in either way, he should not again be set at liberty until after examination and unanimous recommendation for release by a competent board of physicians appointed for that purpose. If this plan were adopted rigidly, most "homicidal cranks" would remain incarcerated for life after having committed murder. For my own part I should like to see capital punishment abolished altogether. This done, the plan I have advocated would possess the still further advantage that the plea of insanity would not be apt to be urged without cause when it was known that the defendant, whether sane or insane, would be incarcerated for life if it were shown that he actually committed homicide. In stating this I do not wish to be understood as saying that the plea of insanity is to-day unduly urged. Indeed, I can indorse the position taken by Kiernan,* who, in a very recent article, says: "The enormous number of insane in penitentiaries proves that the plea of insanity, in place of being abused, is too rarely used."

Under the circumstances I have named, attorneys would largely be relieved of the odium which now attaches to setting up the plea of insanity, with the certainty in almost every case of being accused by certain newspapers of setting up what they are pleased to call the "insanity dodge."

---

*Alienist and Neurologist. April, 1895. P. 140.

